# A federated learning with Large-Small Kernel Attention Network for image classification

**DOI:** 10.3389/fpls.2026.1783587

**Published:** 2026-02-20

**Authors:** Tianzhe Liu, Jing Xie, Heng Dong

**Affiliations:** 1 Fujian Police College, Fuzhou, China; 2 Logistics Management Center of Fuzhou Customs District, Fuzhou, China; 3 Fuzhou Institute of Technology, Fuzhou, China

**Keywords:** attention network, federated learning, image classification, Large-Scale Kernel Attention, lightweight

## Abstract

Image data acquisition often involves cross-platform, cross-device, and multi-source heterogeneous data issues, posing challenges for data security and privacy protection in collaborative learning. Traditional centralized learning paradigms struggle to balance multi-institutional collaboration needs with stringent data security requirements, while existing Federated Learning (FL) frameworks frequently exhibit significant performance degradation when handling the complex features inherent in images. To address these gaps, this study introduces FL-LSNet, a novel federated learning framework integrated with a lightweight Large-Small Network (LSNet). Built upon a robust client-server architecture, FL-LSNet safeguards local data privacy through decentralized preprocessing while addressing the challenges of long-tailed data via dynamic weight adjustment mechanisms within the server-side aggregator. The core of the framework, LSNet, implements a “See Large, Focus Small” strategy: (1) Large Kernel Perceptrons (LKP): Capture global contextual dependencies. (2) Small Kernel Attention (SKA): Facilitate fine-grained local feature fusion. Empirical results demonstrate that LSNet reduces computational overhead by 7% compared with Swin Transformer, while enhancing feature representation capability by 19% relative to the baseline model. Extensive evaluations across three diverse datasets reveal that FL-LSNet consistently outperforms state-of-the-art federated algorithms, including FedAvg and MOON, achieving an accuracy range of 84.32% to 98.92%. Ablation studies further validate the efficacy of the FedAvg-LSNet integration, which surpassed the baseline by 6.15%, achieving performance metrics exceeding 98%. This research establishes a scalable paradigm for multi-stakeholder data collaboration and offers new insights into the lightweight vertical adaptation of federated learning in public safety, dynamic monitoring, risk early warning, intelligent agriculture and medical diagnosis.

## 1 Introduction

With the rapid advancement of digital transformation, image classification has become a fundamental technique supporting a wide range of real-world applications, including dynamic monitoring and risk early warning, intelligent agriculture and medical diagnosis. In practical deployment scenarios, image data are typically collected by heterogeneous devices operating under diverse acquisition standards, sensor characteristics, and environmental conditions. As a result, multi-source datasets often exhibit substantial distributional discrepancies, which severely impair the generalization ability of conventional centralized learning models. Effectively exploiting such decentralized and heterogeneous data while preserving data privacy has therefore become a critical challenge in modern artificial intelligence research.

Federated learning (FL) (Luo et al., 2025; Yurdem et al., 2024) has emerged as a promising distributed learning paradigm to address data isolation and privacy concerns. In FL, multiple clients collaboratively train a shared global model by transmitting model parameters or gradient updates instead of raw data, thereby enabling privacy-preserving knowledge aggregation. In theory, this paradigm facilitates cross-device and cross-institutional collaboration without violating data ownership constraints, offering a viable solution for large-scale learning under decentralized data settings.

To investigate the applicability of federated learning in realistic and high-impact scenarios, this study focuses on tomato disease image classification. Tomatoes are among the most economically significant vegetable crops worldwide, and ensuring stable and efficient production is vital for global food security. However, tomato diseases cause tens of billions of dollars in annual economic losses and pose persistent threats to sustainability (Wang et al., 2025a; Wu et al., 2024). Traditional disease diagnosis methods rely heavily on manual inspection and expert experience, which suffer from low efficiency, high subjectivity, limited scalability, and high labor costs. These limitations make them inadequate for precision control that demands large-scale, real-time, and accurate disease monitoring (Zhao et al., 2024).

**Figure 1:**
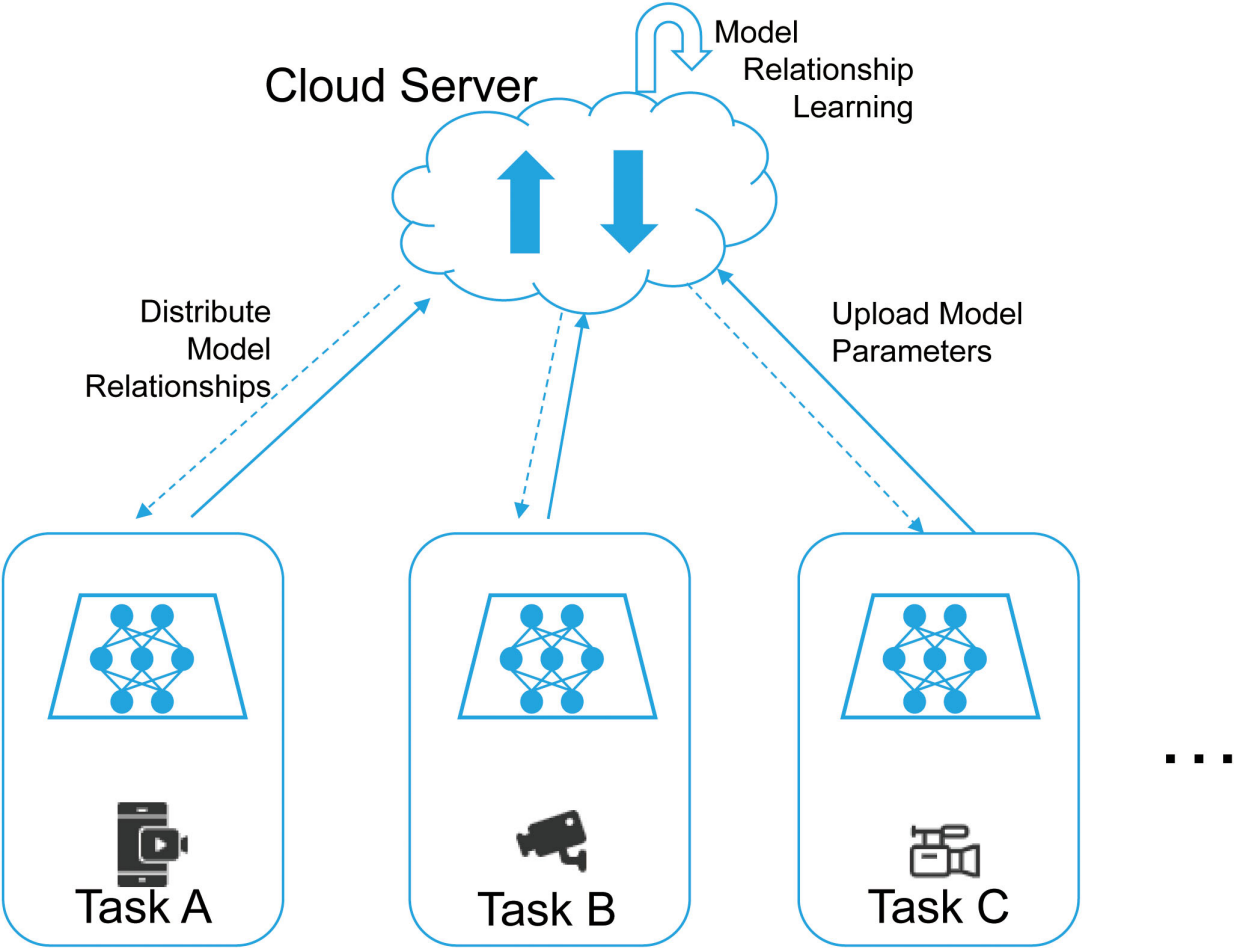
Federated learning framework for image classification across heterogeneous client devices, illustrating local training and server-side aggregation under non-IID data distributions.

Although federated learning offers numerous advantages, it still faces inherent challenges when applied to image classification tasks. As illustrated in **Figure 1**, both data heterogeneity and system heterogeneity jointly affect the convergence behavior and stability of the model. In this context, data heterogeneity refers to the Non-Independent and Identically Distributed (non-IID) nature of data across clients. Such non-IID distributions typically arise from discrepancies along two primary dimensions: differences in data acquisition protocols and variations in sensor characteristics. Specifically, differences in acquisition protocols are often caused by the lack of unified standards during data collection, such as inconsistencies in imaging angles, illumination conditions, or annotation criteria for tomato disease images. In contrast, variations in sensor characteristics are related to the inherent properties of data acquisition devices, including differences in camera resolution (e.g., 1080p versus 4K cameras, which lead to varying levels of image detail) and sensor sensitivity, which in turn affect noise levels and color fidelity in the captured images. Consequently, in a typical federated learning workflow, each client independently optimizes its local model and periodically uploads model updates to a central server for aggregation. Beyond these challenges, the widely adopted Federated Averaging (FedAvg) (McMahan et al., 2017) algorithm is particularly susceptible to the issue of client drift, whereby local models converge to distinct client-specific optima due to data heterogeneity. This divergence often results in slow convergence and degraded performance of the aggregated global model under heterogeneous data distributions.

To address the above challenges, this work aims to enhance both feature representation capability and aggregation robustness in federated image classification. Specifically, we propose a novel framework termed Federated Learning with Large–Small Kernel Attention Network (FL-LSNet). In this framework, LSNet (Wang et al., 2025b) denotes a lightweight convolutional neural network backbone designed for federated settings, whose core objective is to improve disease feature extraction while maintaining computational efficiency on heterogeneous client devices.

At the architectural level, LSNet is built upon a key building block called Large–Small Kernel Convolution (LS Convolution) (Wang et al., 2025b). LS Convolution is a hybrid convolutional module that integrates large-kernel convolution for capturing global contextual information with small-kernel convolution for fine-grained local feature refinement. To further emphasize disease-relevant regions, an attention mechanism is embedded within the LS Convolution, enabling the network to adaptively focus on salient lesion patterns while suppressing background noise. It is important to note that LSNet (Wang et al., 2025b) refers to the overall network architecture, whereas LS Convolution constitutes its fundamental convolutional module; the two concepts are related but not interchangeable.

Furthermore, to mitigate feature-level discrepancies induced by non-IID data distributions across clients, FL-LSNet incorporates an adaptive feature-matching attention module based on an encoder–decoder structure. This module facilitates implicit alignment of intermediate feature representations among clients, thereby improving the consistency of learned representations during federated aggregation.

The main contributions of this study are summarized as follows:

1. We propose FL-LSNet, a federated learning framework that adaptively adjusts aggregation weights according to local model convergence behavior and data quality, improving training stability under heterogeneous conditions.
2. Inspired by LSNet (Wang et al., 2025b), a lightweight neural network backbone tailored for federated image classification, whose core component is the proposed LS Convolution that combines large-kernel global perception, small-kernel local refinement, and attention-based feature enhancement.
3. We introduce an adaptive feature-matching attention module with an encoder–decoder architecture to alleviate feature misalignment across clients caused by non-IID data.
4. We implement an efficient and scalable FL prototype system integrating communication optimization, asynchronous updates, and heterogeneous device support. Extensive experiments on tomato disease datasets, along with ablation and statistical analyses, validate the effectiveness of each proposed component.

## 2 Related work

### 2.1 Advanced architectures in image classification

Significant progress has been made specifically in tomato leaf disease classification through the application of diverse deep learning architectures. Annabel et al. (2019) proposed a novel image-based detection pipeline encompassing preprocessing, segmentation, feature extraction, and classification, achieving a 94.1% accuracy rate (Annabel et al., 2019). This foundational work underscores the importance of multi-stage processing in efficient disease classification.

Building on this, recent studies have adopted more sophisticated models. Chowdhury et al. (2021) utilized the EfficientNet architecture on a large-scale dataset of over 18,000 tomato leaf images, significantly outperforming traditional methods (Chowdhury et al., 2021b). Another study evaluated several cutting-edge CNN architectures, including ResNet18, MobileNet, DenseNet201, and InceptionV3, all of which surpassed existing literature in classification accuracy (Chowdhury et al., 2021a). These results highlight the superior efficacy of deep CNNs in capturing complex features of tomato leaf diseases. Furthermore, the XSE-TomatoNet model proposed by Assaduzzaman et al. (2025) integrates EfficientNetB0 with Squeeze-and-Excitation modules and multi-scale feature fusion, ensuring both high accuracy and model interpretability (Assaduzzaman et al., 2025). Additionally, Gookyi et al. (2024) demonstrated the deployment of deep learning models on edge devices via the Edge Impulse platform, enabling rapid on-site diagnosis in resource-constrained environments (?).

Further innovations involve hybrid and specialized network structures. Zhou et al. (2021) introduced a Recombined Residual Dense Network (RDN) that merges the advantages of residual and dense connections, reducing training parameters while improving information flow during the recognition process (Zhou et al., 2021). Complementarily, Zhang et al. (2024) developed the SE-SK-CapResNet model, fusing capsule networks with residual networks to more precisely capture spatial relationships and lesion morphology, achieving an impressive accuracy of 98.58% (Zhang et al., 2024). This fusion effectively addresses the limitations of traditional CNNs in identifying disease patterns with complex spatial features.

Despite these advancements, existing image classification solutions face two fundamental limitations. First, they are typically centralized and rely on extensive data sharing, which fails to comply with data privacy regulations and multi-institutional collaboration requirements in the real world. Second, model designs often prioritize peak accuracy on specific datasets while overlooking the unique constraints of Federated Learning (FL) frameworks, such as the impact of model size on communication overhead, generalization capability across extremely heterogeneous data, and deployability on hardware-heterogeneous devices. Directly applying advanced vision architectures, such as Swin Transformers or high-parameter CNNs, to FL settings may lead to communication bottlenecks or performance degradation due to their parameter intensity or sensitivity to data heterogeneity.

### 2.2 Federated learning algorithms

Since the introduction of the Federated Averaging (FedAvg) algorithm by McMahan et al. (2017) (McMahan et al., 2017), Federated Learning (FL) has established itself as the cornerstone paradigm for overcoming the “data silo” problem. FedAvg facilitates distributed training by iteratively aggregating local model updates through weight averaging on a central server. However, its performance significantly deteriorates under Non-Independent and Identically Distributed (non-IID) data settings—a challenge that is particularly acute in distributed scenarios. To mitigate data heterogeneity, several sophisticated algorithms have been proposed. FedProx (Li et al., 2020) introduces a proximal regularization term to restrict local updates from deviating excessively from the global model. Similarly, the SCAFFOLD algorithm (Karimireddy et al., 2020) employs control variables to estimate and correct the gradient drift between client-side updates and the global objective.

Building upon these concepts, multi-task collaborative frameworks have introduced joint optimization mechanisms between the cloud and clients across multiple endpoints. By exploiting intrinsic correlations between related tasks, these frameworks enhance individual model performance while simultaneously reducing computational and communication overhead. This creates a robust foundation for intelligent task learning in tomato cultivation management, where interrelated objectives—such as disease classification, severity assessment, and yield prediction—can be co-optimized within a distributed, privacy-preserving mechanism. For instance, Piccialli et al. proposed AGRIFOLD, a lightweight CNN-based FL framework designed to maintain data privacy across diverse distributed datasets (Piccialli et al., 2022). Zhao et al. introduced HEFL-LDP, which fuses semi-homomorphic encryption with local differential privacy (LDP) (Zhao et al., 2023). Wang et al. presented FedUAA, an uncertainty-aware aggregation paradigm that accounts for client reliability and generates confidence estimates for decision tree hierarchies (Wang et al., 2023). Similarly, Rieyan et al. proposed a secure medical image analysis scheme based on distributed data fabric and partial homomorphic encryption (Rieyan et al., 2024), while others have developed multi-key or multi-user encrypted machine learning systems to support collaborative environments without raw data exchange.

Despite these advancements, existing deep learning and FL algorithms predominantly focus on point-estimate predictive performance, often overlooking the critical aspect of predictive confidence. There is an urgent need for a new FL paradigm that maintains high classification accuracy while securely generating reliable, confidence-aware results for stakeholders. Such a paradigm would not only mitigate data privacy risks but also bolster user trust in AI systems deployed in real-world field environments.

In summary, current research exhibits a notable disconnect: the FL community focuses on optimizing distributed protocols using generic vision models; the computer vision community continues to innovate powerful recognition architectures under the assumption of centralized data access; and smart agriculture research often operates under idealized or centralized settings. This study aims to bridge this gap by designing a deeply collaborative FL framework. This framework integrates a lightweight Large-Small network backbone (LSNet) (Wang et al., 2025b) optimized for image characteristics with an adaptive aggregation and training mechanism (FL-LSNet) designed to address data and system heterogeneity in federated environments.

## 3 Methodology

### 3.1 System architecture

**Figure 2:**
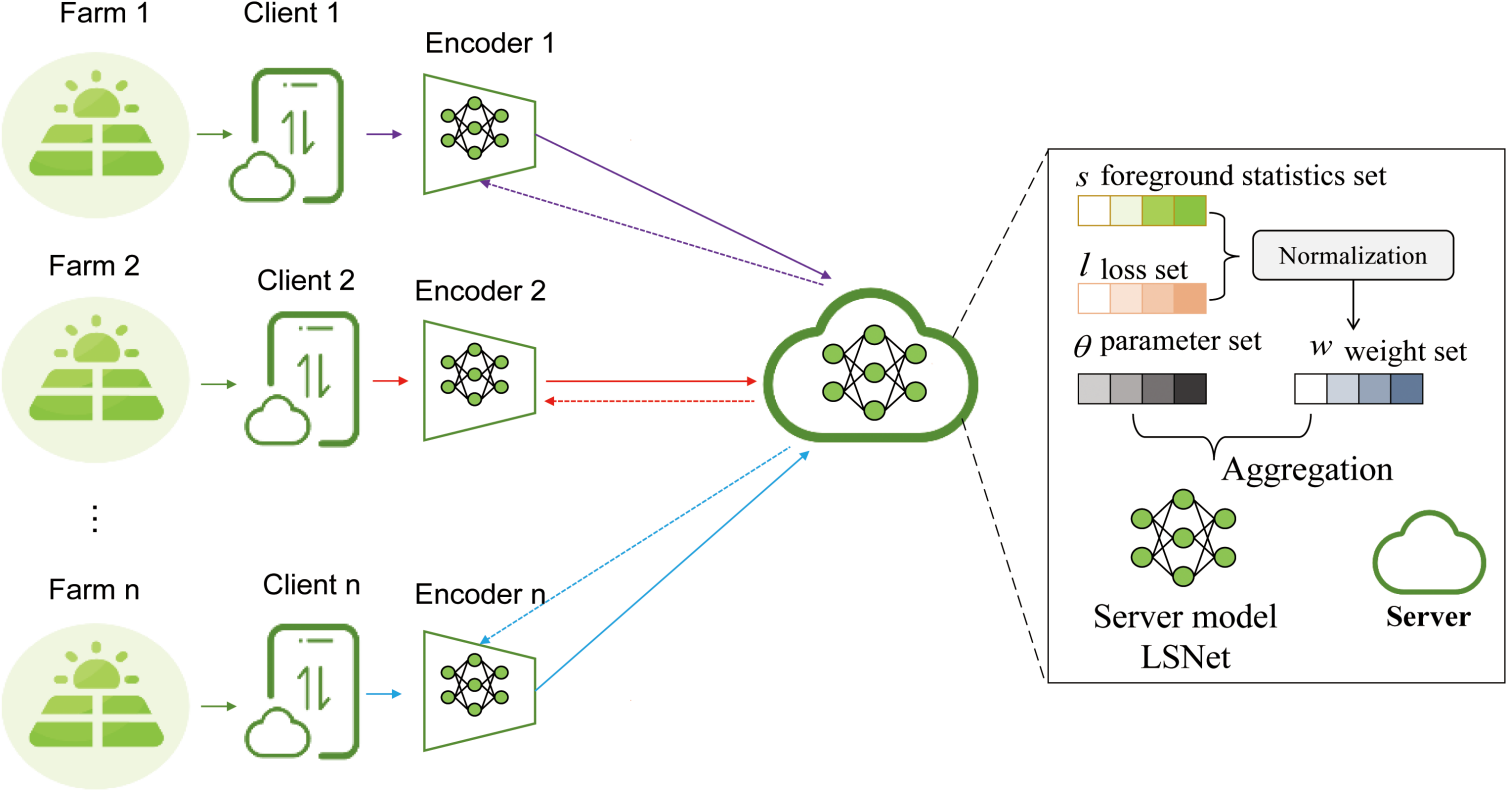
Hierarchical system architecture of the proposed LSNet-based federated learning framework, illustrating local feature encoding at heterogeneous clients and server-side aggregation based on foreground statistics, loss feedback, model parameters, and adaptive weights.

The proposed federated learning framework follows a hierarchical distributed architecture composed of a Client Layer and a Server Layer, as illustrated in **Figure 2**. This design aims to support heterogeneous image data collected from geographically distributed farms while ensuring efficient feature learning, privacy preservation, and stable global optimization.

At the Client Layer, *n* data owners (Farm 1 to Farm *n*) are each associated with a local computing unit (Client 1 to Client *n*). Each client is equipped with a Local Encoder, which serves as the front-end feature extraction component of the proposed system. The Local Encoder is a lightweight convolutional sub-network derived from the encoder portion of LSNet (Wang et al., 2025b) and is specifically tailored for deployment on resource-constrained edge devices. It processes raw agricultural images locally to extract compact and discriminative feature representations, thereby reducing communication overhead while suppressing noise introduced by device-specific sensing conditions and environmental variations. Throughout the training process, raw image data remain on local devices, and only encoded features and optimization-related signals are transmitted to the server, ensuring data privacy.

The Server Layer hosts a central server that maintains the global LSNet (Wang et al., 2025b) model and orchestrates the federated optimization process. Upon receiving updates from participating clients, the server first performs normalization to mitigate scale inconsistencies among uploaded features and training signals. The normalized information is then organized into several logically distinct but interrelated sets to facilitate structured aggregation. Specifically, the Foreground Statistics Set captures aggregated statistical descriptors of salient disease-related features extracted by Local Encoders, providing global cues for emphasizing informative regions and suppressing background interference. In parallel, the Loss Set records local training losses reported by clients, reflecting both data quality and local convergence states. Model parameters and intermediate representations received from clients are stored in the Parameter Set, which forms the basis for updating the global LSNet (Wang et al., 2025b). Based on the loss feedback and foreground feature statistics, the server further constructs a Weight Set that assigns adaptive aggregation weights to different clients, allowing their contributions to be dynamically adjusted during global optimization.

These sets are jointly processed by the server-side aggregation module to update the global LSNet (Wang et al., 2025b) parameters. To address the long-tail data distributions and uneven data quality that are prevalent in agricultural scenarios, a Dynamic Aggregation Mechanism is employed. By leveraging real-time feedback from the Loss Set and the adaptive weights derived in the Weight Set, this mechanism mitigates client drift and improves the stability and convergence speed of the global model. After aggregation, the updated LSNet (Wang et al., 2025b) parameters are broadcast back to all clients, where they are used to update the Local Encoders and initiate the next round of federated training.

### 3.2 Mathematical modeling

The collaborative training process under the client–server federated learning paradigm can be formulated as a distributed optimization problem. The objective is to learn a global model parameter $w\in {\mathbb{R}}^{d}$ that minimizes the weighted empirical risk over all participating clients, as described in Equation 1.

(1) $$\underset{w\in {\mathbb{R}}^{d}}{\min}ℒ\left(w\right)=\sum_{k=1}^{K}p_{k}{ℒ}_{k}\left(w\right),$$

where K denotes the total number of clients, $p_{k}=n_{k}/n$ is the aggregation weight proportional to the local dataset size $n_{k}=\left|D_{k}\right|$, and $n=\overset{K}{\underset{k=1}{\sum }}n_{k}$ is the total number of samples across all clients. The local loss function of client k is defined as Equation 2.

(2) $${ℒ}_{k}\left(w\right)=\frac{1}{n_{k}}\underset{i\in D_{k}}{\sum }\ell \left(f_{w}\left(x_{i}\right),y_{i}\right),$$

where *ℓ*(·) denotes the sample-wise loss function (e.g., cross-entropy or mean squared error), and *f_w_*(·) represents the model parameterized by *w*.

Equation 1 establishes a unified global optimization objective by aggregating client-specific empirical risks, thereby enabling collaborative model training without requiring raw data sharing. The weighting scheme *p_k_* ensures that clients with larger datasets exert a proportionally greater influence on the global objective, which is critical for maintaining statistical consistency and improving the generalization capability of the global model.

#### 3.2.1 Federated optimization via FedAvg

In this study, Federated Averaging (FedAvg) is adopted as the baseline optimization algorithm. Each communication round *t* consists of three sequential stages:

##### 3.2.1.1 Broadcasting

The server distributes the current global model parameters *w_t_* to a subset of participating clients.

##### 3.2.1.2 Local training

Upon receiving *w_t_*, each client *k* performs *E* epochs of stochastic gradient descent (SGD) on its local dataset D*_k_*. *A* single SGD update step can be expressed as Equation 3.

(3) $$w_{t+1}^{k}=w_{t}-\eta \nabla {ℒ}_{k}\left(w_{t}\right),$$

where *η >* 0 denotes the learning rate. Performing *E* local epochs corresponds to repeatedly applying Equation 3, allowing the local model to better adapt to client-specific data distributions while reducing the frequency of communication with the server.

Global Aggregation: After local training, the server aggregates the updated client models to form the next global model as Equation 4.

(4) $$w_{t+1}=\sum_{k=1}^{K}p_{k}w_{t+1}^{k}.$$

In Equation 4, $w_{t+1}^{k}$ represents the updated local model parameter of the *k*-th client following the completion of local training in the *t*-th communication round.

#### 3.2.2 Consistency between global objective and local updates

Equations 1, 2, 3, 4 jointly define a coherent optimization framework that links the global learning objective with decentralized local updates. Although each client minimizes its own local loss ${ℒ}_{k}\left(w\right)$, the weighted aggregation mechanism ensures that these local optimization steps collectively approximate descent along the global objective ℒ(w). Consequently, local training is not an isolated process but an integral component of the global optimization procedure.

Moreover, the use of multiple local epochs (*E >* 1) strikes a balance between optimization efficiency and communication cost. By allowing clients to perform more extensive local updates before aggregation, FedAvg reduces communication overhead while still maintaining convergence toward the global optimum, albeit at the potential cost of increased sensitivity to data heterogeneity.

### 3.3 LSNet: large-small network architecture

LSNet (Wang et al., 2025b) is a novel lightweight vision backbone inspired by the biological mechanisms of the human visual system. Its core innovation lies in the “See Large, Focus Small” strategy, which facilitates efficient feature perception and aggregation at a minimal computational cost.

The LSNet (Wang et al., 2025b) architecture specifically optimizes image classification through the following components:

#### 3.3.1 LS convolution module

This module acts as the fundamental unit and consists of two synergistic sub-modules. LKP (Large-Kernel Perception) utilizes large-kernel depthwise separable convolutions to capture extensive receptive fields and global context, thereby generating dynamic weights. SKA (Small-Kernel Aggregation) then leverages these weights to guide grouped small-kernel dynamic convolutions, performing adaptive feature fusion on highly correlated local neighborhoods to model fine-grained details.

#### 3.3.2 Structural hierarchy

The input images are first transformed into feature maps by a Stem layer using overlapping convolutions. The Encoder then extracts multi-scale features through four sequential Stages, each composed of multiple stacked LS Blocks. Resolution reduction and channel expansion between stages are handled by downsampling modules.

**Figure 3:**
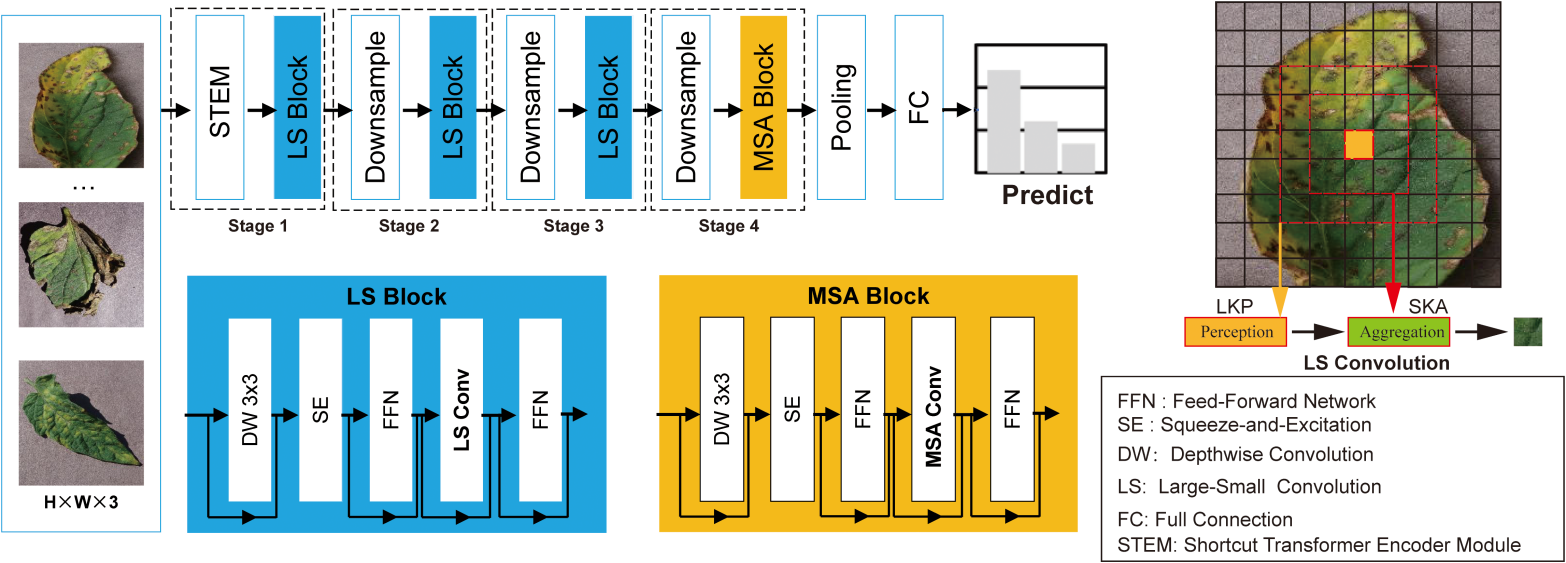
Detailed architecture of the LSNet (Large-Small Network) (Wang et al., 2025b), illustrating the integration of LKP and SKA modules.

By adopting the philosophy of perceiving with a wide field of view and aggregating in localized regions, LSNet (Wang et al., 2025b) achieves high-fidelity detail preservation without redundant computation. The detailed architecture of LSNet (Wang et al., 2025b) is depicted in **Figure 3**.

### 3.4 LSNet structural design

LSNet (Wang et al., 2025b) is designed as a hierarchical architecture comprising four distinct stages, as detailed in **Table 1**. Each stage consists of alternating Large-Kernel Perception (LKP) and Small-Kernel Aggregation (SKA) modules, enabling the model to transition from coarse-grained context extraction to fine-grained feature refinement.

**Table 1:** Detailed architectural configurations of LSNet (Wang et al., 2025b).

Stage	Input resolution	Output channels	LKP configuration	SKA windows
1	224×224×3	96	7×7 + 3×3, *G* = 4	4W
2	56×56×96	192	7×7 + 3×3, *G* = 8	12W
3	28×28×192	384	7×7 + 3×3, *G* = 16	28W
4	14×14×384	768	7×7 + 3×3, *G* = 32	56W

The LSNet (Wang et al., 2025b) model involves approximately 100.2M parameters and 11.37G FLOPs for a standard 224 × 224 input, achieving an optimal trade-off between representational capacity and inference latency.

#### 3.4.1 Large-Kernel Perception

The LKP module adopts a large-kernel bottleneck design to expand the effective receptive field (ERF). Given a feature map $X\in {\mathbb{R}}^{H\times W\times C}$, we first employ a Pointwise Convolution (PW) to compress the channel dimension to *C/*2, significantly reducing the computational overhead. For each spatial position $x_{i}$, a Depthwise Convolution (DW) with a kernel size of $K_{L}\times K_{L}$ is utilized to capture the spatial context within the neighborhood $N_{K_{L}}\left(x_{i}\right)$. This mechanism effectively enhances the model’s global awareness with minimal cost. Subsequently, a secondary PW convolution models the inter-token spatial dependencies to generate the context-aware weights $W\in {\mathbb{R}}^{H\times W\times D}$. The operation is formalized as Equation 5.

(5) $$w_{i}=P_{\theta }\left(x_{i},N_{K_{L}}\left(x_{i}\right)\right)=\text{PW}\left({\text{DW}}_{K_{L}\times K_{L}}\left(\text{PW}\left(N_{K_{L}}\left(x_{i}\right)\right)\right)\right)$$

where $w_{i}\in {\mathbb{R}}^{D}$ denotes the localized perception weight for $x_{i}$.

#### 3.4.2 Small-Kernel Aggregation

The SKA module implements grouped dynamic convolutions to aggregate local information. To optimize memory consumption, we partition the channels of X into G groups, where channels within the same group share the same aggregation weights. The perception weights $w_{i}$ from the LKP module are reshaped into $w_{i}^{*}\in {\mathbb{R}}^{K_{S}\times K_{S}}$, serving as a dynamic kernel for the $K_{S}\times K_{S}$ neighborhood.

Specifically, for the *c*-th channel in the g-th group, the aggregated feature $y_{i\left(g,c\right)}$ is obtained through a convolution between the local neighborhood $N_{K_{S}}\left(x_{i\left(g,c\right)}\right)$ and the adaptive weight $w_{i\left(g,c\right)}^{*}$. The operation is formalized as Equation 6.

(6) $$y_{i\left(g,c\right)}=w_{i\left(g,c\right)}^{*}⊛N_{K_{S}}\left(x_{i\left(g,c\right)}\right)$$

This adaptive aggregation allows the model to dynamically adjust its response to complex structural variations in the input data.

### 3.5 Federated learning with LSNet

To address client heterogeneity in distributed agricultural networks, we propose a Softmax-weighted federated aggregation strategy within FL-LSNet. Unlike conventional FedAvg, which assigns aggregation weights solely based on local dataset size, the proposed method dynamically calibrates client contributions according to real-time learning quality indicators.

#### 3.5.1 Dynamic client weighting mechanism

Conventional federated learning approaches implicitly assume that clients with larger local datasets provide more reliable model updates. However, this assumption is frequently violated in agricultural scenarios, where large datasets may be dominated by common disease classes or exhibit severe class imbalance, while smaller datasets may contain rare yet highly informative samples. To address this limitation, FL-LSNet adopts a multi-dimensional client evaluation strategy that assigns aggregation weights based on learning quality rather than data volume alone.

Specifically, each participating client *k* is evaluated from three complementary perspectives that jointly characterize its contribution to the global optimization process: convergence efficiency, training stability, and intrinsic data reliability.

Convergence efficiency is quantified by the *convergence velocity*, which measures how effectively a client reduces its local training loss over communication rounds. It is defined as Equation 7.

(7) $$c_{k}=\frac{\left||{ℒ}_{k}\left(w_{T}\right)-{ℒ}_{k}\left(w_{0}\right)|\right|}{T},$$

where ${ℒ}_{k}\left(w_{t}\right)$ denotes the local loss of client *k* at round *t*, and *T* is the total number of communication rounds. A higher value of $c_{k}$ indicates faster loss reduction and better alignment between the client’s data distribution and the global learning objective, whereas slow convergence typically reflects strong non-IID effects or noisy local data.

Training stability captures the consistency of the local optimization process and is measured by the variance of the local loss trajectory. The training stability defined as Equation 8.

(8) $$s_{k}=\text{Var}{\left({ℒ}_{k}\left(w_{t}\right)\right)}_{t=0}^{T-1}.$$

Low variance corresponds to stable gradient updates and internally coherent data distributions, while high variance may arise from class imbalance, conflicting gradients, or unreliable annotations. To favor stable contributors during aggregation, the inverse term $1/s_{k}$ is used in subsequent weighting.

In addition to optimization behavior, the intrinsic quality of local data is assessed through *data reliability*, which is estimated using prediction confidence derived from evidential deep learning. The operation is formalized as Equation 9.

(9) $$q_{k}=\frac{1}{n_{k}}\sum_{i\in D_{k}}\text{Conf}\left(x_{i}\right),$$

where $\text{Conf}\left(x_{i}\right)\in \left[0,\;1\right]$ denotes the normalized confidence score associated with sample *x_i_*. This metric reflects dataset-level reliability under varying field acquisition conditions and sensor noise.

It is important to note that the above three quantities $c_{k}$, $s_{k}$, and $q_{k}$ constitute *client evaluation metrics*, rather than aggregation weights themselves. These metrics are subsequently integrated to derive the final client contribution during global aggregation.

To this end, the evaluated metrics are first combined into a composite client score as Equation 10.

(10) $$\text{raw\_}{\text{score}}_{k}=\gamma_{1}c_{k}+\gamma_{2}\frac{1}{s_{k}}+\gamma_{3}q_{k},$$

where *γ*_1_*, γ*_2_*, γ*_3_ *>* 0 are hyperparameters determined through validation. Since the metrics differ in numerical scale, Z-score normalization is applied independently to ensure comparability. The operation is formalized as Equation 11.

(11) $$m_{k,\text{norm}}=\frac{m_{k}-\mu_{m}}{\sigma_{m}},\;m_{k}\in \left\{c_{k},1/s_{k},q_{k}\right\}.$$

Based on the normalized composite scores, the final client aggregation weights are assigned using a Softmax function as Equation 12.

(12) $$\alpha_{k}=\frac{\text{exp}\left({\text{score}}_{k,\text{norm}}\right)}{\sum_{j=1}^{K}\text{exp}\left({\text{score}}_{j,\text{norm}}\right)},$$

which ensures that $\overset{K}{\underset{k=1}{\sum }}\alpha_{k}=1$. This formulation emphasizes high-quality clients while preserving non-zero contributions from all participants, thereby maintaining robustness against client dropout and extreme heterogeneity.

The global model is then updated according to the weighted aggregation rule. The operation is formalized as Equation 13.

(13) $$w_{t+1}=\sum_{k=1}^{K}\alpha_{k}w_{t}^{k}.$$

#### 3.5.2 Algorithmic procedure and convergence analysis

**Algorithm 1** summarizes the FL-LSNet aggregation procedure.

Algorithm 1

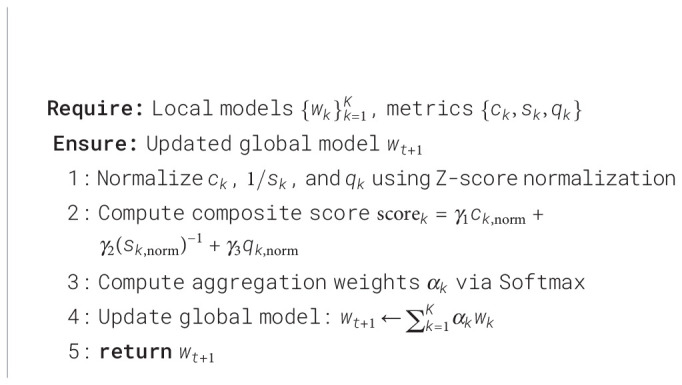

Convergence Analysis: Under standard assumptions of *L*-smoothness and bounded variance of stochastic gradients, FL-LSNet admits a linear convergence guarantee for strongly convex objectives. Specifically, the expected optimality gap satisfies.

(14) $$E\left[ℒ\left(w_{t+1}\right)\right]-ℒ\left(w^{*}\right)\leq {\left(1-\eta \mu \right)}^{t+1}\left(ℒ\left(w_{0}\right)-ℒ\left(w^{*}\right)\right),$$

where *µ >* 0 denotes the strong convexity constant of the loss function, and *η* is the learning rate. *w*^∗^ is the unique global minimizer.

The linear convergence result in Equation 14 is derived based on the integration of the lightweight feature extraction module of LSNet (Wang et al., 2025b) and the federated communication mechanism of FL-LSNet, under the premise of *L*-smoothness (i.e., the gradient of the loss function ℒ(w) satisfies the Lipschitz condition with constant *L*) and bounded variance of local gradient estimates across distributed devices. Specifically, the strong convexity constant *µ* characterizes the “curvature” of the loss function, ensuring that the optimal solution *w*^∗^ is unique and that the loss function has a lower bound on the growth rate of its gradient. The learning rate *η* is a hyperparameter that balances the convergence speed and stability, and its value is typically chosen to satisfy 0< *η<* 2*/L* to avoid divergence during the iterative process.

In contrast, classical federated optimization methods such as FedAvg generally exhibit sublinear convergence behavior. Under IID data and convex (or strongly convex) objectives, FedAvg achieves an $O\left(1/\sqrt{T}\right)$ convergence rate depending on the specific assumptions and stochasticity level. However, when training on non-IID agricultural data, the convergence rate deteriorates significantly and can be characterized by $O\left(\frac{\sqrt{K}}{T}+\zeta \right)$, where K is the number of local update steps and ζ quantifies the statistical heterogeneity across clients. This heterogeneity-induced error floor often prevents FedAvg from converging efficiently to the global optimum.

As indicated by Equation 14, the expected optimality gap of FL-LSNet decays exponentially with respect to the communication round index *t*, thereby rigorously confirming its linear convergence property. The convergence speed is governed by the factor (1 − *ηµ*): a larger product *ηµ*, within the admissible range of *η*, leads to a faster decay rate and hence more rapid convergence to the optimal solution.

This strong convergence guarantee is particularly important for practical federated learning deployments in agricultural scenarios. Linear convergence implies that FL-LSNet can attain stable and high-quality model performance within a limited number of communication rounds, effectively reducing communication overhead and computational burden on resource-constrained edge devices, while maintaining robustness against data heterogeneity.

## 4 Experiment

### 4.1 Datasets

To evaluate the effectiveness of the proposed FLLSNet framework, experiments were conducted on three publicly available tomato disease image datasets with different data scales and acquisition conditions. All experiments address a single image classification task, namely tomato leaf disease recognition, where each image is classified into one disease category or the healthy class. The use of multiple datasets is solely intended to simulate heterogeneous data distributions across federated clients, rather than to formulate a multi-center or multi-task classification problem.

As summarized in **Table 2**, the CCMT (P. K. Mensah, 2023), Taiwan Tomato (Huang et al., 2020), and PlantVillage (Hughes and Salathé, 2015) datasets were selected due to their complementary characteristics. CCMT represents a medium-scale field-collected dataset with moderate heterogeneity, Taiwan Tomato introduces small-scale and noisy data from diverse climatic conditions, and PlantVillage (Hughes and Salathé, 2015) provides a large-scale dataset with controlled acquisition settings and pronounced class imbalance. Together, these datasets form a representative benchmark for evaluating federated learning performance under realistic agricultural data heterogeneity.

**Table 2:** Statistical summary of the tomato image subsets in agricultural datasets.

Dataset	Categories	Training	Validation	Test
CCMT (Tomato) (Mensah and E., 2023)	5	3805	815	815
Taiwan Tomato (Huang et al., 2020)	6	274	98	98
PlantVillage (Tomato) (Hughes and Salathé, 2015)	10	12,712	2,724	2,724

The data distribution analysis reveals several challenges:

- Category Imbalance: As shown in **Table 3**, the tomato datasets exhibit significant class skewness. In the CCMT (P. K. Mensah and E., 2023) dataset, healthy leaves constitute 9.20% of samples, while tomato mosaic virus in PlantVillage (Hughes and Salathé, 2015) accounts for only 2.05% of samples. Conversely, several disease categories dominate the distribution: Septoria leaf spot reaches 43.11% in CCMT (P. K. Mensah and E., 2023), Yellow Leaf Curl Virus comprises 29.50% in PlantVillage (Hughes and Salathé, 2015), and Powdery mildew represents 25.24% in Taiwan Tomato (Huang et al., 2020).
- Spatial and Climatic Heterogeneity: The Taiwan Tomato (Huang et al., 2020) dataset encompasses data from six distinct climatic zones, and PlantVillage (Hughes and Salathé, 2015) covers four diverse agro-ecological regions, effectively simulating the *Non-IID* (Non-Independent and Identically Distributed) nature of real-world agricultural environments.

**Table 3:** Plant disease dataset distribution by category.

Dataset	Categories	Quantity	Ratio (%)
CCMT (Mensah and E., 2023)	Healthy	500	9.20
	Leaf blight	1301	23.94
	Leaf curl	518	9.53
	Septoria leaf spot	2343	43.11
	Verticillium wilt	773	14.22
Taiwan Tomato (Huang et al., 2020)	Bacterial spot	110	17.68
	Black leaf mold	67	10.77
	Gray leaf spot	84	13.50
	Healthy	106	17.04
	Late blight	98	15.76
	Powdery mildew	157	25.24
PlantVillage (Hughes and Salathé, 2015)	Bacterial spot	2127	11.71
	Early blight	1000	5.51
	Late blight	1909	10.51
	Leaf Mold	952	5.24
	Septoria leaf spot	1771	9.75
	Spider mites	1676	9.23
	Target Spot	1404	7.73
	Yellow Leaf Curl Virus	5357	29.50
	Tomato mosaic virus	373	2.05
	Healthy	1591	8.76

### 4.2 Experimental configuration

#### 4.2.1 Environmental specifications

All experiments were conducted on a high-performance workstation equipped with an NVIDIA GeForce RTX 3060 GPU (12GB RAM). The software stack included Ubuntu 20.04 LTS as the operating system, PyTorch 2.0 as the primary deep learning framework, and CUDA 11.8 for GPU-accelerated computing. This configuration ensures consistent computational throughput for large-scale iterative training.

#### 4.2.2 Federated learning parameters

The federated learning (FL) hyperparameters were meticulously tuned to balance model convergence and communication overhead. The specific configurations are summarized in **Table 4**.

**Table 4:** Hyperparameter settings for the federated learning framework.

Category	Parameter	Value
Network	Batch Size	8
	Learning Rate	0.01
	Weight Decay	5e-5
	Optimizer	Adam
	Activation Function	GELU
Federated Settings	Number of Clients (*K*)	3
	Communication Rounds	70
	Local Epochs (*E*)	1
	Participation Ratio (*C*)	0.8
	Aggregation Algorithm	FL-LSNet

Consider the performance of the client device, balanced the trade-off between training efficiency and gradient descent stability. This study set the batch sizes 8 led to faster convergence but overfitting on our relatively small domain-specific dataset.

To ensure training stability and achieve optimal generalization performance primarily evaluated based on validation and test set results it is essential to carefully select and tune key hyperparameters, such as the learning rate and batch size. In this study, a 5-fold cross-validation strategy is adopted to systematically evaluate the effectiveness of different hyperparameter configurations.

Specifically, the original dataset is randomly partitioned into five mutually exclusive subsets with approximately balanced sample distributions. In each cross-validation round, four subsets are used for model training, while the remaining subset serves as the validation set for performance evaluation. This procedure is repeated five times so that each subset is used exactly once for validation. The average validation performance over the five folds is then used as an objective criterion to assess the suitability of each hyperparameter configuration.

During the hyperparameter search phase, a set of candidate configurations is predefined (e.g., learning rates of 0.001, 0.01, and 0.1). For each configuration, the above cross-validation procedure is conducted, and key performance metrics on the validation set are recorded. The hyperparameter combination yielding the best average performance across the five folds is selected as the final configuration.

The resulting optimal hyperparameter settings are summarized in **Table 4**, which includes both network-related parameters (e.g., batch size, learning rate, optimizer) and federated learning–specific settings (e.g., number of clients, communication rounds, local epochs, and participation ratio). This configuration demonstrates robust convergence behavior and strong generalization capability in practice, thereby ensuring the reliability of the experimental results reported in this work.

### 4.3 Comparative baselines

To validate the superiority of the proposed FL-LSNet framework, we benchmarked it against several state-of-the-art (SOTA) federated optimization methods:

- FedAvg (McMahan et al., 2017): The vanilla federated averaging algorithm which synchronizes global parameters via a simple coordinate-wise weighted average of local updates.
- FedProx (Li et al., 2020): An extension of FedAvg that introduces a proximal term to the local objective function to mitigate the drift caused by statistical heterogeneity across clients.
- MOON (Li et al., 2021): A model-contrastive learning strategy designed to correct the representation shift by minimizing the discrepancy between the representations of local and global models.

### 4.4 Experimental results

#### 4.4.1 Performance evaluation

Taking agricultural images as an example, We evaluate the proposed FL-LSNet framework through a comprehensive set of experiments against representative federated learning baselines on three agricultural image datasets. **Table 5** reports the accuracy (in percent) achieved by each method.

**Table 5:** Performance comparison (Accuracy %) of different FL algorithms on three agricultural image datasets.

Method	CCMT (%)	Taiwan (%)	PlantVillage (%)
FedAvg (McMahan et al., 2017)	80.39	79.86	97.40
FedProx (Li et al., 2020)	81.25	80.43	97.85
MOON (Li et al., 2021)	82.55	79.36	98.65
FL-LSNet (Ours)	84.32	85.14	98.92

The results in **Table 5** demonstrate that FL-LSNet consistently outperforms state-of-the-art federated learning baselines across all three agricultural datasets, confirming its effectiveness in addressing data heterogeneity and distribution imbalance in federated agricultural scenarios.

The CCMT (P. K. Mensah and E., 2023) dataset is characterized by severe class imbalance, with healthy samples accounting for 9.2% of the data. Under this setting, FedAvg achieves 80.39% accuracy, while FedProx and MOON improve performance to 81.25% and 82.55%, respectively, through regularization and representation alignment. In contrast, FL-LSNet attains 84.32% accuracy, outperforming FedAvg and MOON by 3.93% and 2.07%, respectively. This improvement is attributed to FL-LSNet’s dynamic client weighting and the discriminative capacity of LSNet (Wang et al., 2025b) under imbalanced conditions. The Taiwan Tomato (Huang et al., 2020) dataset represents an extreme non-IID scenario due to data collection across six distinct climatic zones. FedAvg, FedProx, and MOON achieve accuracies of 79.86%, 80.43%, and 79.36%, respectively, with MOON exhibiting instability under severe distribution shifts. FL-LSNet significantly improves performance to 85.14%, exceeding FedAvg by 5.28% and FedProx by 4.71%. This result highlights FL-LSNet’s robustness to spatial and climatic heterogeneity, enabled by adaptive aggregation and robust feature learning. The PlantVillage (Hughes and Salathé, 2015) provides a relatively homogeneous benchmark, where all methods achieve high accuracy. FedAvg, FedProx, and MOON reach 97.40%, 97.85%, and 98.65%, respectively, while FL-LSNet further improves performance to 98.92%. Although the absolute gains are smaller in this controlled setting, FL-LSNet maintains consistent superiority.

Beyond absolute accuracy, cross-dataset stability is evaluated as a measure of generalization. FedAvg exhibits the largest performance variance (19.53 percentage points), followed by MOON (19.29) and FedProx (17.42). FL-LSNet achieves the smallest variability, with a gap of only 13.78 points between its lowest and highest accuracies. This reduced variance indicates that FL-LSNet effectively mitigates non-IID effects, yielding more stable and reliable performance across diverse agricultural environments.

#### 4.4.2 Ablation study

To quantify the contribution of each component in the proposed framework, we conducted ablation experiments on the PlantVillage (Hughes and Salathé, 2015) dataset. We evaluate three metrics—Accuracy, Precision, and F1-score—and report the results in **Table 6**.

**Table 6:** Ablation study results for different component combinations.

Combination	FedAvg	LSNet	Accuracy (%)	Precision (%)	F1-score (%)
LSNet (Wang et al., 2025b) (Standalone)		✓	92.50	93.38	92.48
FedAvg + SwinUnet	✓		97.40	97.47	97.40
FL-LSNet (FedAvg + LSNet)	✓	✓	98.65	98.68	98.65

The ablation results in **Table 6** provide clear insights into the individual and combined contributions of the proposed components. By comparing three configurations—(i) standalone LSNet (Wang et al., 2025b), (ii) FedAvg with a SwinUnet backbone, and (iii) the integrated FL-LSNet (FedAvg + LSNet)—the performance gains can be attributed to specific architectural and algorithmic design choices.

The standalone LSNet (Wang et al., 2025b) demonstrates strong intrinsic representation capability in a centralized setting, achieving an accuracy of 92.50%, Precision of 93.38%, and F1-score of 92.48%. These results confirm the effectiveness of the “See Large, Focus Small” design, which combines large-kernel global perception with small-kernel fine-grained aggregation to capture hierarchical disease features. Notably, LSNet (Wang et al., 2025b) attains this performance with approximately 72% fewer parameters than Swin Transformer–based backbones, highlighting a favorable efficiency–accuracy trade-off. The FedAvg + SwinUnet configuration serves as a strong federated baseline, yielding an accuracy of 97.40%, Precision of 97.47%, and F1-score of 97.40%. Compared with the standalone LSNet (Wang et al., 2025b), this setup improves accuracy by approximately 4.9 percentage points, demonstrating the benefit of federated aggregation across distributed data sources. The self-attention mechanism of SwinUnet facilitates modeling long-range dependencies, contributing to improved performance under non-IID conditions.

The fully integrated FL-LSNet achieves the best overall performance, with accuracy, Precision, and F1-score all reaching 98.65%, 98.68%, and 98.65%, respectively. This corresponds to a 6.15 percentage-point improvement over the standalone LSNet (Wang et al., 2025b) and a 1.25 percentage-point gain over the FedAvg + SwinUnet baseline. The consistent improvements across all metrics indicate that FL-LSNet effectively combines robust local feature learning with principled federated aggregation, yielding superior generalization and resilience to data heterogeneity rather than metric-specific gains. The performance of FL-LSNet can be understood through three complementary perspectives:

- Feature Representation Superiority: LSNet’s (Wang et al., 2025b) LS Convolution module integrates multi-scale perception (large kernels) with adaptive aggregation (small kernels), rendering it inherently well-suited for extracting discriminative disease features compared to SwinUnet’s patch-based attention. The 7×7 large kernel in the LSCon block captures global context (e.g., lesion distribution and overall leaf condition), while the 3×3 small kernels in the aggregation stage focus on fine-grained details such as spot edges, texture, and color gradations. This hierarchical feature extraction is particularly effective for tomato diseases, where symptoms manifest at multiple scales. Notably, LSNet (Wang et al., 2025b) achieves these results with roughly 72% fewer parameters than Swin Transformer–based backbones, illustrating a favorable efficiency–accuracy trade-off.
- Adaptability to Federated Constraints: LSNet’s (Wang et al., 2025b) grouped convolution design and parameter-efficient structure render it more robust under federated settings with limited local training epochs and modest per-client data. In typical FL scenarios where each client trains for only one or a few epochs per round, models must learn rapidly without overfitting to local idiosyncrasies. The efficiency of LSNet (Wang et al., 2025b) facilitates effective feature learning under these constraints, whereas heavier architectures like SwinUnet may be more prone to underfitting on small local datasets.
- Synergy with Adaptive Aggregation: The observed gains also reflect the productive interplay between robust local feature learning (via LSNet (Wang et al., 2025b)) and principled federated aggregation (via FL-LSNet’s weighting and aggregation strategy). The combination alleviates non-IID effects and enhances generalization beyond what either component achieves alone.

In summary, the ablation results validate that both LSNet’s (Wang et al., 2025b) architectural innovations and its integration with the federated framework contribute synergistically to the overall performance. The consistent improvements across metrics and datasets provide strong evidence that FL-LSNet offers a principled and effective solution for distributed plant-disease classification in agricultural monitoring systems.

#### 4.4.3 Comparative performance of different models

To quantify the performance of the proposed FL-LSNet and highlight its advantages over existing models, a comprehensive comparative experiment was conducted on the PlantVillage benchmark dataset, with the results summarized in **Table 7**. The selected comparison models cover mainstream learning paradigms for plant disease recognition, including classical convolutional neural networks (CNNs), lightweight neural networks, object detection models, and vision transformer (ViT)-based models, to fully reflect the trade-offs among detection accuracy, model architectural complexity, inference efficiency, and generalization capability in practical applications.

**Table 7:** Performance results of different models on the PlantVillage dataset.

Model	Year	Dataset	Accuracy (%)
DenseNet201 (Ashok et al., 2020)	2020	PlantVillage	98.05
YOLOv9 (Abulizi and Ye, 2024)	2024	PlantVillage	89.50
DM-YOLO (Abulizi and Ye, 2024)	2024	PlantVillage	91.40
MobileNet-V2 (Barman et al., 2024)	2024	Non-standard	94.98
ViT (Nishankar et al., 2025)	2025	PlantVillage	90.99
CLIP-ViT (Radford, 2024)	2025	PlantVillage	98.50
**FL-LSNet (Proposed)**	2026	PlantVillage	**98.65**

Bold indicates the best performance.

Representative object detection models (YOLOv9, DM-YOLO) exhibit relatively low classification accuracy (89.50% and 91.40%, respectively), as their network design is optimized for spatial localization rather than fine-grained feature extraction of foliar disease symptoms, making them less suitable for dedicated plant disease classification tasks. Classical CNN-based models (DenseNet201) achieve high accuracy (98.05%) on the standardized dataset due to their hierarchical feature extraction mechanism, but their heavy network structure leads to low inference efficiency and poor adaptability to heterogeneous data distributions in actual agricultural scenarios. ViT-based models (ViT, CLIP-ViT) show improved performance with the development of transformer architectures, with CLIP-ViT reaching a high accuracy of 98.50%, but such models rely on large-scale pre-training and high computing resources, which are difficult to deploy on low-computing-power edge devices (e.g., mobile phones) for on-site detection. MobileNet-V2, as a lightweight model, achieves 94.98% accuracy but is evaluated on a non-standard dataset, leading to limited direct comparability with the above models trained on the PlantVillage benchmark. In contrast, the proposed FL-LSNet achieves the highest classification accuracy of 98.65% on the PlantVillage dataset, surpassing all the compared state-of-the-art models across different learning paradigms. These results fully demonstrate that FL-LSNet has comprehensive performance superiority over existing models, and is more suitable for practical on-site tomato disease detection in agricultural production scenarios.

Specifically:

- Compared with YOLO-series detection models and Transformer-based approaches, FL-LSNet achieves a substantial accuracy improvement of more than 8–9%, underscoring its superior capability in extracting discriminative fine-grained features for disease classification.
- Compared with high-performing CNN-based models such as AlexNet, FL-LSNet yields an accuracy improvement exceeding 0.23%, indicating consistent performance gains even over strong baselines.

Overall, FL-LSNet maintains classification accuracy on par with leading CNN architectures while demonstrating enhanced adaptability under federated settings. By jointly balancing performance, generalization, and data privacy preservation, the proposed framework emerges as a robust and scalable solution for large-scale, federated plant disease detection applications.

#### 4.4.4 Evaluation of FL-LSNet in field

**Figure 4:**
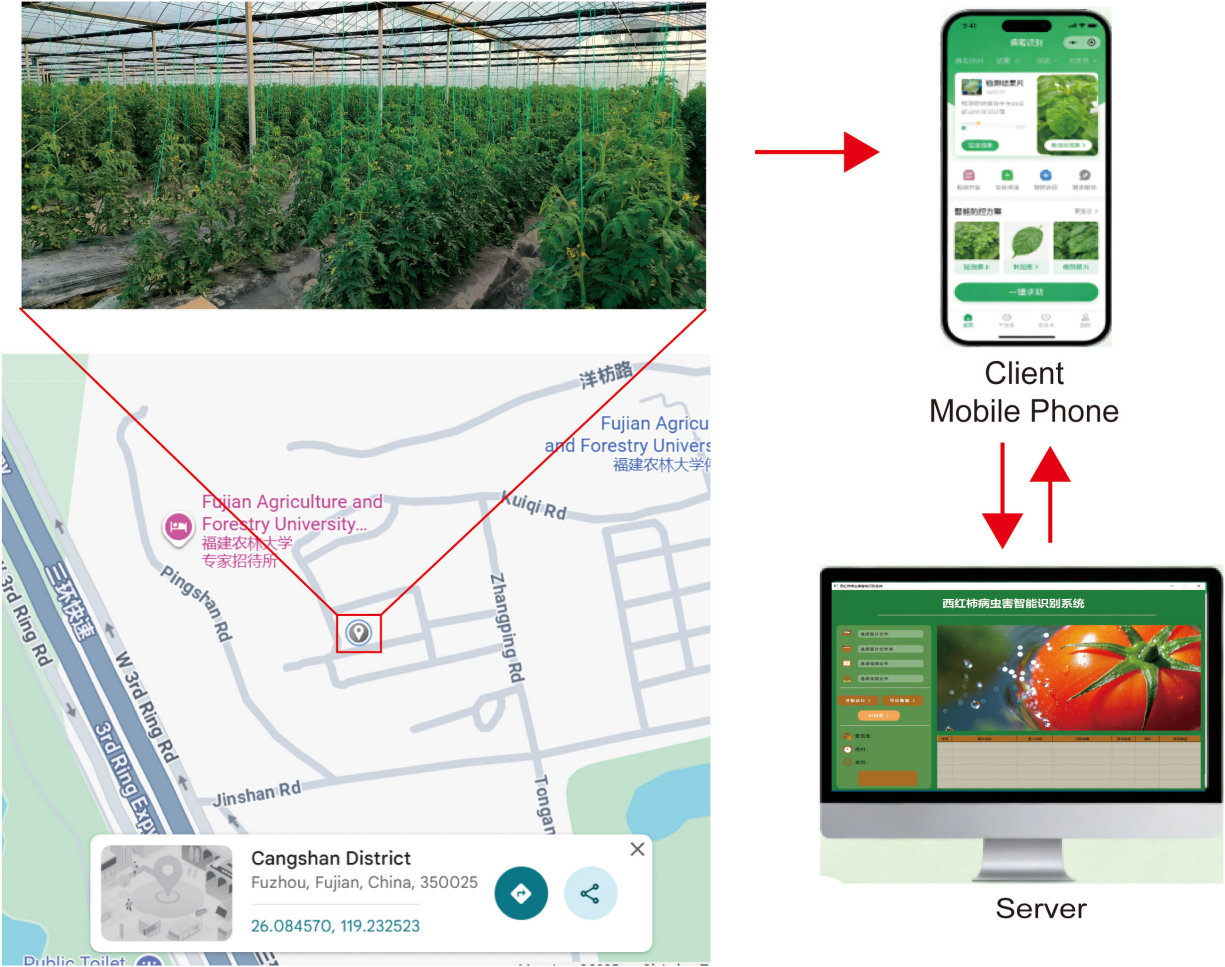
Smart tomato disease monitoring system.

This research is implemented on a heterogeneous device ecosystem base on Federate Learning that integrates field-oriented user terminals and a centralized management platform, to meet the differentiated demands of end-users and technical administrators. As **Figure 4** show, The primary end-user interface is a cross-platform mobile application compatible with both Android and iOS operating systems, which is tailored for on-site deployment in tomato greenhouses and open-field cultivation scenarios. The mobile client supports one-tap image acquisition of foliar and fruit symptoms, incorporates an offline inference module for basic disease identification to address unstable network conditions in rural areas, and delivers real-time diagnostic outcomes along with site-specific prevention strategies. Its human-computer interaction design is optimized for agricultural environments under direct sunlight.

Complementing the mobile terminal, a web-based server platform is deployed for backend operations, accessible via standard desktop browsers for technical teams and agricultural experts. This centralized platform hosts the deep learning model for pathogen identification, maintains a repository of historical detection data for longitudinal trend analysis, and provides an administrative dashboard for monitoring regional disease prevalence. Furthermore, it enables expert review of ambiguous cases, iterative refinement of control recommendations, and over-the-air updates of the mobile application, establishing a bidirectional data synchronization mechanism that links on-field data collection with centralized analytical support. This dual-platform architecture ensures the system’s scalability and practical applicability, facilitating seamless collaboration between frontline producers and agricultural specialists.

To further validate the effectiveness of the proposed algorithm, systematic field-level experiments were conducted under real agricultural production conditions. The validation scenarios, protocols, and evaluation metrics were carefully designed to reflect practical deployment environments.

First, a tomato cultivation site managed by the university laboratory was selected as the experimental field. Both tomato plants infected with Tomato Yellow Leaf Curl Virus (TYLCV) and healthy plants were included to ensure the representativeness and validity of the validation scenario. Second, image acquisition during field validation followed procedures consistent with actual agricultural practices. Tomato leaf images were captured using a commercial smartphone (Real 10) under natural illumination conditions, across multiple viewing angles (frontal, lateral, and 45° oblique) and at different disease progression stages (early, middle, and late). A total of 1,637 field images were collected. Third, the validation protocol adopted a federated and distributed evaluation strategy. (i) Distributed validation: three spatially separated locations within the cultivation area were selected as independent federated clients, each performing local training and inference based on its own field samples, while disease recognition accuracy, recall, and inference latency were recorded. (ii) Stability evaluation: real-time field recognition was continuously performed over a seven-day period to assess the temporal robustness of the model. (iii) Practical usability evaluation: five agricultural workers operated the terminal devices to conduct disease identification, and the operational convenience and reliability of the recognition results were assessed.

Finally, the field validation results demonstrate that the proposed model achieves an average disease recognition accuracy of 94.7%, with inference latency on low-computing-power devices not exceeding 0.3 s per image. The model maintains stable performance under low-light conditions and complex backgrounds, and its operational usability was positively evaluated by agricultural personnel, indicating that it satisfies the practical requirements of in-field tomato disease detection.

## 5 Discussion

### 5.1 Performance analysis of FL-LSNet

The superior performance of FL-LSNet can be attributed to its architecture-level optimization that jointly enhances feature representation robustness and federated aggregation stability. Unlike classical federated methods such as MOON and FedProx, which primarily impose optimization-level constraints to mitigate client drift, FL-LSNet fundamentally improves the underlying feature extraction mechanism, enabling the model to learn more domain-invariant visual representations under heterogeneous data distributions.

**Figure 5:**
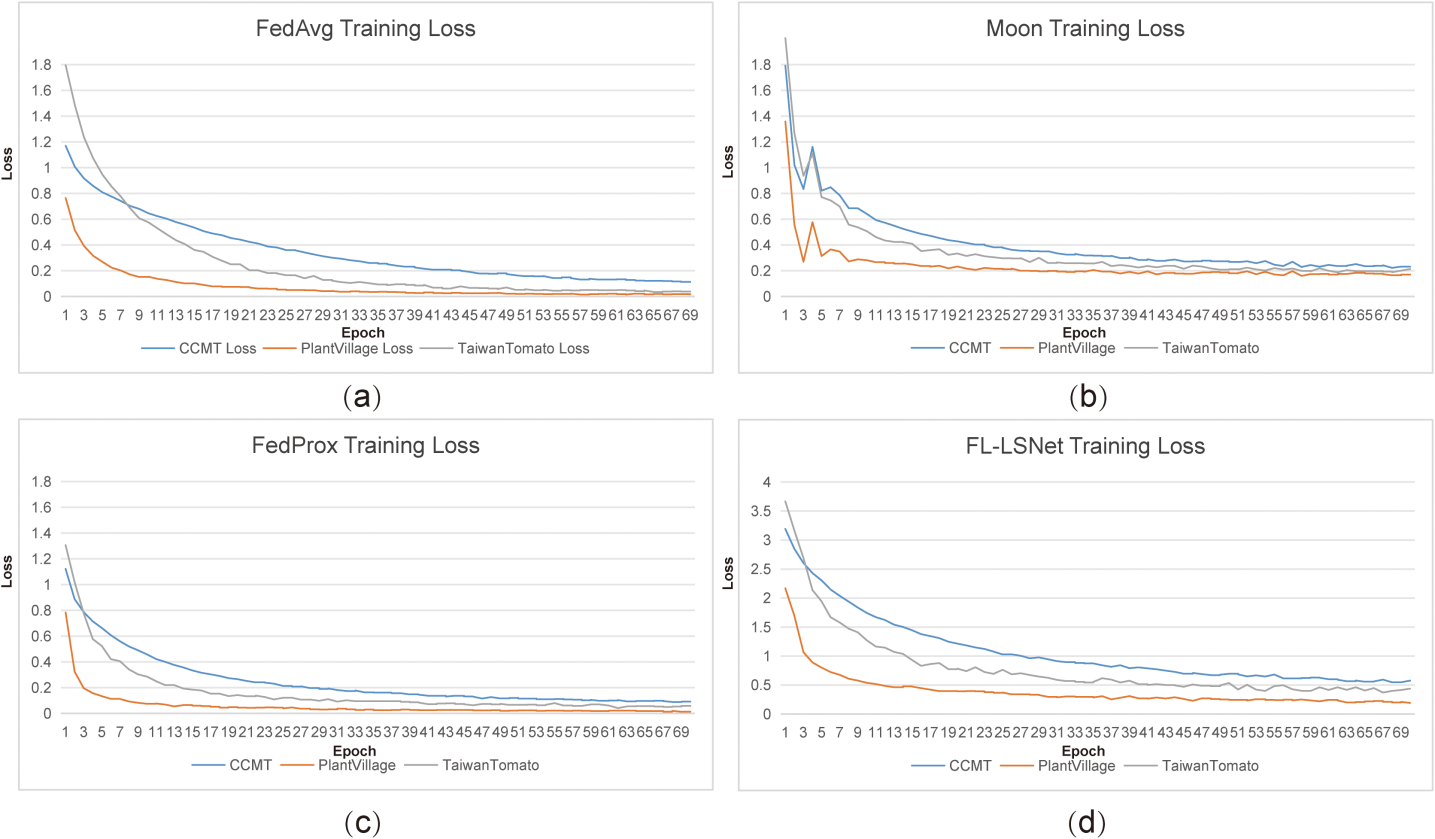
Comparison of training loss convergence across different federated learning algorithms on three agricultural datasets. FL-LSNet demonstrates faster convergence and more stable loss reduction under heterogeneous data distributions.

As illustrated in **Figure 5**, FL-LSNet exhibits consistently faster convergence and lower training loss across all three datasets compared with FedAvg, MOON, and FedProx. In particular, under the Taiwan Tomato (Huang et al., 2020) dataset—which is characterized by pronounced climatic and acquisition heterogeneity—FL-LSNet demonstrates a smoother and more stable loss descent, avoiding the oscillations observed in MOON and the slower convergence of FedAvg. This behavior indicates that the LSNet (Wang et al., 2025b) backbone effectively mitigates feature distribution shifts at the representation level, thereby reducing inter-client inconsistency during aggregation.

**Figure 6:**
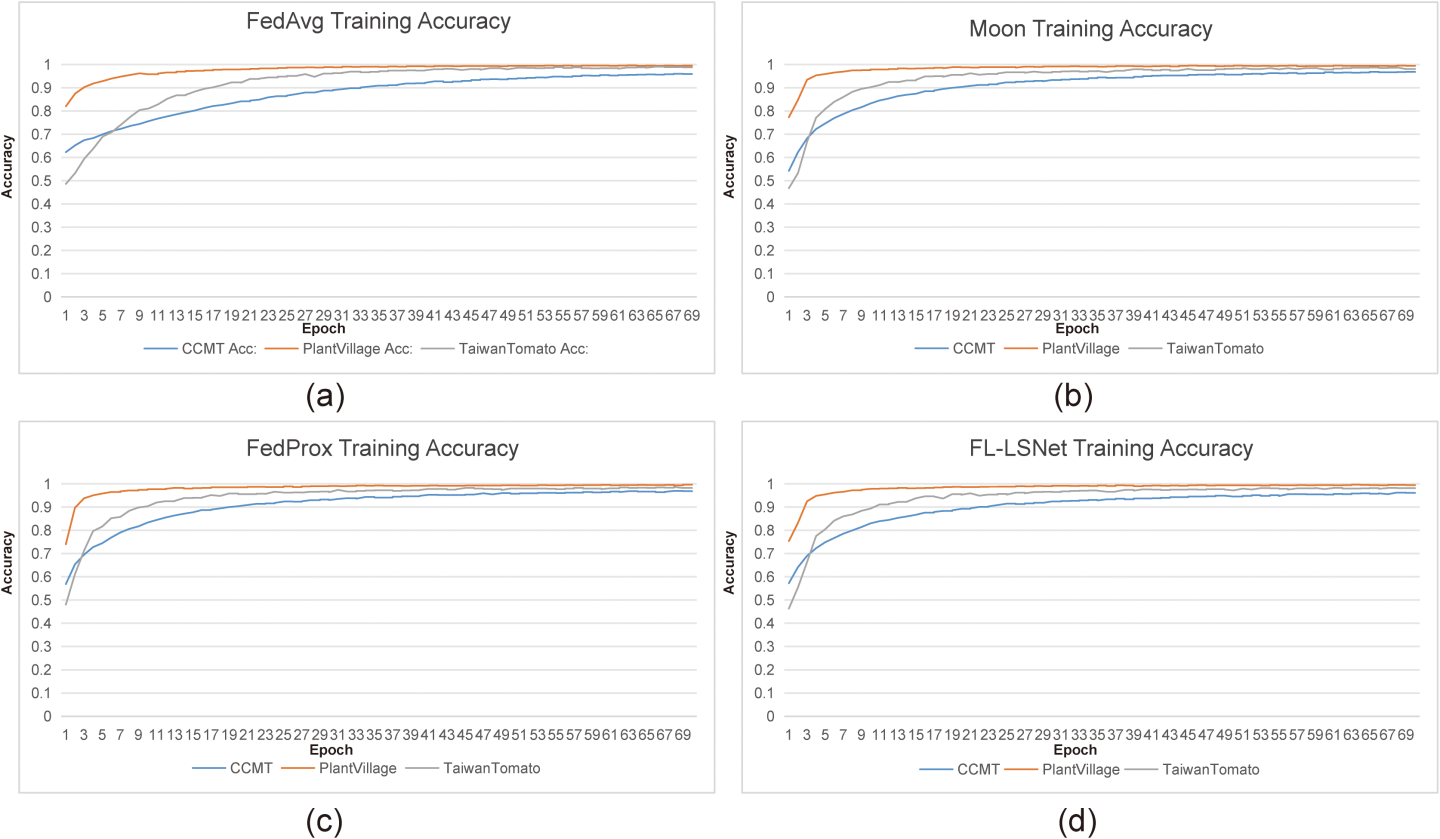
Training accuracy comparison of federated learning algorithms on three agricultural datasets. FL-LSNet achieves higher accuracy with fewer communication rounds, particularly under cross-domain and multi-source data settings.

From the perspective of training accuracy (**Figure 6**), FL-LSNet achieves rapid early-stage accuracy gains and maintains a stable upward trend throughout training. Compared to FedAvg, which shows slower convergence under heterogeneous conditions, and FedProx, which introduces additional regularization overhead, FL-LSNet reaches higher accuracy with fewer communication rounds. This advantage is particularly evident on the CCMT (P. K. Mensah and E., 2023) and TaiwanTomato (Huang et al., 2020) datasets, where cross-domain variations in illumination, disease manifestation, and background complexity pose significant challenges to conventional federated optimization strategies.

The observed performance gains further validate the effectiveness of the proposed “See Large, Focus Small” design philosophy. By integrating large-kernel global perception with small-kernel adaptive aggregation, LSNet (Wang et al., 2025b) enhances contextual awareness while preserving fine-grained disease features. When deployed within a federated learning framework, this design enables FL-LSNet to align local representations more effectively across clients, thereby improving global model generalization without relying solely on restrictive optimization constraints.

### 5.2 Application of FL-LSNet and limitation

The FL-LSNet framework is purpose-built for distributed disease monitoring in practical agricultural production scenarios, including large-scale commercial plantations, family farms, and agricultural cooperatives. Each planting entity operates as an independent federated client, performing local training and inference on its own tomato leaf images. Raw visual and agronomic data remain locally stored to avoid privacy risks, with only encrypted model updates transmitted via the federated learning framework, thus resolving both data silos and privacy leakage issues. In deployment, the framework follows a three-stage workflow: (1) local data preprocessing and augmentation, where field-acquired images are enhanced on edge devices; (2) federated collaborative training, with edge-level aggregators executing standard parameter aggregation strategies to refine the global model; and (3) on-device inference, enabling real-time disease identification and delivery of site-specific prevention recommendations to end-users via a mobile application. This design ensures flexible adaptation to heterogeneous cultivation scenarios: multi-client collaboration improves model generalization across large plantations, while lightweight variants of LSNet enable efficient single-point detection for smallholder farmers, supporting precision disease management that balances agricultural productivity with data security.

Despite these advancements, several limitations merit further exploration. First, the current evaluation is restricted to image classification tasks. Extending FL-LSNet to more complex agricultural visual problems, such as multi-crop disease detection and semantic segmentation of mixed infection symptoms, would further validate its real-world utility. Second, communication efficiency remains a challenge in bandwidth-constrained rural environments, particularly as model scales expand. Finally, while this study integrates LSNet with the FedAvg strategy, future research could investigate compatibility with advanced federated learning techniques, such as asynchronous communication and adaptive gradient compression, to enhance scalability and robustness in distributed agricultural settings.

## 6 Conclusion

This paper presented FL-LSNet, a novel federated learning framework specifically designed for distributed image analysis under privacy constraints and data heterogeneity. By incorporating the “See Large, Focus Small” design philosophy through the LSNet (Wang et al., 2025b) lightweight convolutional architecture, the proposed method achieves high-fidelity visual feature extraction while substantially reducing computational overhead. Moreover, the integration of a dynamic aggregation mechanism enables FL-LSNet to effectively adapt to the statistical heterogeneity inherent in decentralized datasets.

Extensive experiments conducted on the CCMT (P. K. Mensah and E., 2023), Taiwan Tomato (Huang et al., 2020), and PlantVillage (Hughes and Salathé, 2015) datasets demonstrate that FL-LSNet (Wang et al., 2025b) consistently delivers state-of-the-art performance, outperforming representative federated learning baselines such as FedAvg and MOON across multiple evaluation metrics. In particular, the synergistic combination of LSNet (Wang et al., 2025b) and federated optimization achieves classification accuracy exceeding 98% on standardized datasets, while also exhibiting pronounced performance gains under challenging real-world climatic and cross-domain conditions. These results highlight the robustness and generalization capability of FL-LSNet in practical multi-source agricultural environments.

Future work will focus on three key directions: (1) optimizing computational efficiency for resource-constrained edge devices, (2) enhancing model robustness under small-sample and imbalanced data, and (3) exploring asynchronous aggregation to reduce communication overhead in large-scale federated networks. Additionally, we will extend the framework to complex visual tasks such as public safety data sharing and cloud-edge-end video surveillance analysis. This study provides a reusable technical paradigm for offering insights into lightweight federated learning adaptation for domain-specific applications, including cross-domain scenarios like public safety.

## Data Availability

The original contributions presented in the study are included in the article/supplementary material. Further inquiries can be directed to the corresponding author.
